# T-Bet Controls Susceptibility of Mice to *Coxiella burnetii* Infection

**DOI:** 10.3389/fmicb.2020.01546

**Published:** 2020-07-14

**Authors:** Soraya Mezouar, Hubert Lepidi, Ikram Omar Osman, Jean-Pierre Gorvel, Didier Raoult, Jean-Louis Mege, Yassina Bechah

**Affiliations:** ^1^IRD, AP-HM, MEPHI, Aix-Marseille University, Marseille, France; ^2^IHU-Méditerranée Infection, Marseille, France; ^3^CIML, CNRS, INSERM, Aix-Marseille University, Marseille, France; ^4^AP-HM, IHU-Méditerranée Infection, UF Immunologie, Marseille, France; ^5^IRD, AP-HM, VITROME, Aix-Marseille University, Marseille, France; ^6^INSERM, Marseille, France

**Keywords:** *Coxiella burnetii*, T-bet, granulomas, bacteria persistence, interferon-γ

## Abstract

T-bet is a transcription factor known to initiate and coordinate the gene expression program during Th1 differentiation, which is crucial for clearance of intracellular pathogens. Q fever is a worldwide zoonosis caused by *Coxiella burnetii*. This bacterium is transmitted to humans by aerosol. Indeed, the inhibition of the *Coxiella*-specific adaptive Th1 immune response leads to persistent infection and organ injury. How deficiency of T-bet affects host infection by *C. burnetii* has not been investigated. Here, using mice with a deletion of the T-bet gene and an airborne mode of infection to reproduce the natural conditions of *C. burnetii* infection, we show that infected T-bet^–/–^ mice were more affected than wild-type mice. The lack of T-bet leads to defective bacterial control, intense replication, persistent infection, and organ injury manifesting as an increased number of granulomas. The absence of T-bet was also associated with an impaired immune response. Indeed, the production of the immunomodulatory cytokines interleukin (IL)-6 and IL-10 was increased, whereas the expression of microbicidal genes by splenocytes was impaired. Moreover, the absence of T-bet exhibited impaired production of interferon-γ, the principal cytokine released by Th1 effector cells. Thus, our study highlights the key role of T-bet in the control of *C. burnetii* infection in mice and leads to a reappraisal of granulomas in the pathogenesis of Q fever disease.

## Introduction

*Coxiella burnetii* is an airborne intracellular Gram-negative bacterium responsible for severe infections (Stein et al., 2005; Eldin et al., 2017). *C. burnetii* infection, called Q fever, is characterized by a self-limiting episode that may evolve several months or years of a persistent infection with mainly lesions on heart valves (Q fever endocarditis) and vascular tissue (Raoult et al., 1987). The immune status of the host is crucial for the outcome of *C. burnetii* infection. The use of mice models permits a better understanding of the immune response in both acute and persistent forms of the infection. After injection of the bacterium by aerolization to mimic natural infection, *C. burnetii* is found in non-immune tissues, including lung and adipose (Bechah et al., 2014) tissues, and immune lymphoid organs, including spleen (Lockhart et al., 2012; Melenotte et al., 2016a) and lymph nodes (Melenotte et al., 2016b). Histological analysis of murine or human tissues showed that *C. burnetii* resides in granulomas, a collection of immune cells (Meghari et al., 2008; Herndon and Rogers, 2013; Eldin et al., 2017). Using an *in vitro* model of granuloma formation (Mezouar et al., 2019b) in the presence of *C. burnetii*, we previously dissected the role of immune cells involved in this process and showed, for the first time, monocytes recognize bacterial extracts and then recruit T lymphocytes, allowing their differentiation into macrophages and functional T lymphocytes, respectively (Delaby et al., 2010).

To better understand the persistence of *C. burnetii*, numerous studies have investigated myeloid cells known as target cells for *C. burnetii*. Infected monocytes exhibit an M1-type program, a proinflammatory state, that allows bacterial survival without replication (Capo et al., 1999; Dellacasagrande et al., 1999; Mahapatra et al., 2010). In macrophages, *C. burnetii* resides in a late acidic phagosome that is unable to fuse with lysosomes, allowing bacteria to escape its destruction (Heinzen et al., 1996; Romano et al., 2007). More recently, the growth and the survival of *C. burnetii* has been attributed to hypoxia-induced hypoxia-inducible factor 1α (HIF1α) in macrophages (Hayek et al., 2019). *C. burnetii* induces an M2-related program in murine alveolar macrophages, which is highly permissive to *C. burnetii* multiplication (Fernandes et al., 2016). This latter program is characterized by the low production of inflammatory cytokines and the overproduction of immunoregulatory cytokines, such as interleukin (IL)-10, which is associated with persistent infection in tissues from mice overexpressing IL-10 (Meghari et al., 2008) and persistent Q fever in humans (Honstettre et al., 2003). More recently, in intraperitoneal or intratracheal infection of Myd88^–/–^ mice, persistence of *C. burnetii* has been observed in organs with less granulomatous inflammation and decreased expression of several genes involved in the intracellular control of bacteria (Kohl et al., 2019). *C. burnetii* also affects the functions of the dendritic cells (DCs). Indeed, the transcriptomic analysis of myeloid DCs (mDCs), stimulated with *C. burnetii*, reveals subtle alterations in type I IFN signaling, an antiviral pathway (Gorvel et al., 2014). Additionally, *C. burnetii* masks its recognition by DC to prevent their maturation and the secretion of inflammatory cytokines (Shannon et al., 2005). The type I IFN pathway was also found to be associated with infection by *C. burnetii* of plasmacytoid DCs (pDCs) *in vitro*. Interestingly, the number of circulating pDCs is significantly lower in patients with Q fever endocarditis than in controls (Ka et al., 2016).

Studies using murine models have illustrated that intact adaptive immune response is required to control *C. burnetii* infection. Indeed, nude and severe combined immunodeficient mice (SCID) are susceptible to *C. burnetii* infection (Melenotte et al., 2016a; van Schaik et al., 2017), and the reconstitution of SCID mice with CD4^+^ or CD8^+^ T cells restores protective immunity (Read et al., 2010). Mice with knockout in the IFN-γ gene are highly susceptible to *C. burnetii* infection (Andoh et al., 2003). In humans, immunocompromised patients suffer from persistent Q fever more frequently than healthy controls (Eldin et al., 2017). Some studies show that the clinical manifestations of persistent Q fever are associated with an altered Th1 response with a defective production of IFN-γ and an overproduction of IL-10 (Capo et al., 1999; Honstettre et al., 2003). Moreover, peripheral blood mononuclear cells from subjects vaccinated against *C. burnetii* show a state of cellular immunity with production of IFN-γ (Izzo and Marmion, 1993). However, altered cell-mediated immunity with higher IFN-γ production in Q fever fatigue syndrome and chronic Q fever patients compared to seropositive controls was also reported in *ex vivo* stimulated whole blood (Keijmel et al., 2016). Chronic Q fever patients present an intact IFN-γ response and production compared to healthy controls (Schoffelen et al., 2013, 2017). These patients presented low IL-2 production and polymorphisms in the IL-12p40 gene (Schoffelen et al., 2014, 2017).

T-box expressed in T cells (T-bet), a transcription factor expressed in numerous hematopoietic cells, is essential to initiate the gene expression program that leads to the elimination of pathogens during the induction of type 1 T helper (Th1) response and is required for optimal production of IFN-γ (Szabo, 2002). How T-bet deficiency affects host infection with *C. burnetii* has not been studied. In this report, we show that T-bet-deficient mice are much more susceptible to *C. burnetii* infection than wild-type (WT) mice. Furthermore, the loss of T-bet leads to an increase in the number of copies of *C. burnetii* DNA in the investigated tissues and an increase in the total number of granulomatous inflammatory lesions in liver and lungs. Using isolated splenocytes from infected mice, we show that the expression of microbicidal genes (encoding perforin, granzyme B, and IFN-γ) and the production of protective IFN-γ were decreased while the production of immunoregulatory cytokines (IL-10 and IL-6) was found to be increased. Taken together, these results show that T-bet controls *C. burnetii* infection in mice through the modulation of the specific immune response and offers new perspectives on the role of granuloma in the persistence of *C. burnetii*.

## Materials and Methods

### Mice and Bacteria

Age (6–8 weeks) and gender (female) matched C57BL/6 WT (Charles River laboratories) and C57BL/6 T-bet-deficient mice (T-bet^–/–^) (Peng et al., 2002) (provided by Dr. J-PG, Centre d’Immunologie de Marseille-Luminy, Marseille, France) were used. The experimental protocol (reference APAFIS #2016101209473180) was approved by the Ethics Committee “C2EA-14,” of Aix-Marseille University, France, and the French Ministry of National Education, Higher Education and Research.

*Coxiella burnetii* Guiana strain (MST17) was cultured in Vero cells as previously described (Bechah et al., 2014). Briefly, cell monolayers were infected for 7–10 days. Cells were sonicated, and bacteria were collected, purified, and titrated as previously described (Bechah et al., 2014). Bacterial viability was assessed using the LIVE/DEAD BacLight kit (Molecular Probes). All the experiments were carried out in a biosafety level 3 laboratory (IHU Mediterranean infection, Marseille, France).

### Mice Infection

Wild-type and T-bet^–/–^ mice were exposed to *C. burnetii* aerosols using the inhalation exposure system “whole-body” type A4224 (Glas-Col LLC, Terre Haute, United States) as previously described (Bechah and Raoult, 2017). Briefly, mice were placed in dedicated cages and transferred to the device for aerosolization. Bacterial suspension (5 × 10^7^) or phosphate-buffered saline (PBS, used as control) were introduced into the venture vial to generate aerosols. This procedure was realized for 1 h and repeated twice. After exposure to *C. burnetii* aerosols, no death or signs of illness/discomfort were observed in WT and T-bet^–/–^ mice up to 22 days postinfection (PI). Blood samples were collected by orbital puncture at different times postinfection (PI) for serology as previously reported (Meghari et al., 2008). Then, mice were sacrificed at 7, 15, and 22 days PI, and spleen, lungs, liver, visceral adipose tissue (AT), tracheal, cervical lymph node (CLN), and lymph node (TLN) were collected and conserved at −80°C or fixed in 5% buffered formalin for DNA extraction or histological studies, respectively.

### Histological Analysis

Fixed tissue biopsies with formalin at 5% were embedded in paraffin and serial sections (3 μm) were performed for hematoxylin-eosin-saffron staining and immunohistochemical analysis. First, the number of granulomas, which are defined as collections of 10 or more macrophages and lymphocytes (Meghari et al., 2008) was determined after a complete optical examination of at least three sections of tissue from each organ using the image analyzer SAMBA 2005 (SAMBA Technologies, Alcatel TITN, Grenoble, France). Results are expressed as the number of granulomas found per surface unit (mm^2^). Second, the presence of bacteria within tissues was determined using rabbit anti-*C. burnetii* antibodies (Abs, 1:2000 dilution) and revealed using a Ventana Benchmark autostainer (Ventana Medical Systems, Inc., Tucson, AZ, United States) as previously described (Bechah et al., 2014).

### Bone Marrow–Derived Macrophages and Splenocytes Culture

Bone marrow cells and splenocytes were collected from femurs (uninfected) and spleen (infected), respectively (Meghari et al., 2008; Bechah and Raoult, 2017).

Briefly, bone marrow cells were isolated from femurs from healthy WT and T-bet^–/–^ mice and cultured in RPMI 1640 containing 25 mM HEPES, 10% fetal bovine serum (FBS), 100 U/ml penicillin, 100 μg/ml streptomycin, and 15% of L-cell conditioned medium rich in macrophage-colony-stimulated. After 7 days of culture, more than 95% of cells were macrophages (bone marrow–derived macrophages, BMDMs) as determined by the morphological criteria and flow cytometry analysis using anti-CD68 monoclonal Abs (data not shown).

Splenocytes were isolated from WT and T-bet^–/–^ mice at different times postinfection as previously reported (Bechah et al., 2010) and were then stored at −80°C.

### Determination of *C. burnetii* Infection

For the molecular detection of *C. burnetii*, 2 × 10^5^ BMDMs were infected with *C. burnetii* (50 bacteria per cell) for 4 h, washed to eliminate unbound organisms (which correspond to day 0 PI), and cultured in RPMI 1640 containing HEPES and FBS for 12 days. DNA from infected BMDMs or tissue biopsies was extracted using a QIAamp Tissue Kit (Qiagen) as previously described (Mezouar et al., 2019d). qPCR was performed using the CFX96 qPCR Detection System (Bio-Rad, France) and carried out with DNA extract and specific primers and probe targeting a fragment of the *C. burnetii* 16S DNA gene. The selected primers and probe were F (5′-ACGGGTGAGTAATGCGTAG-G-3′) R (5′-GCTGATCGTCCTCTCA-GACC-3′) and 6-FAM-GCAAAGCGGGGGATCTTCGG-TAMR, respectively, and the results expressed as cycle threshold (Ct) values for infected BMDMs and DNA copy for tissue biopsies (Mezouar et al., 2019a). Indeed, Ct values of 32.6, 29.4, 25.9, and 22.2 correspond to 5 × 10^2^, 5 × 10^3^, 5 × 10^4^, and 5 × 10^5^ copies of DNA, respectively. The samples were considered positive when the qPCR Ct was <36.

The presence of bacteria within BMDM cells was also revealed using immunofluorescence as previously reported (Bechah et al., 2014). Briefly, infected BMDMs cultured on glass coverslips were fixed with 3% paraformaldehyde and permeabilized with 0.1% Triton X-100. After washing, cells were incubated with rabbit Abs directed against *C. burnetii* (1:500 dilution) for 30 min and then with Alexa 555-conjugated F(ab’) anti-rabbit IgG (Molecular Probes) (1:500 dilution) and bodipy phalloidin (Alexa 488) (Life Technologies) to label filamentous actin (F-actin). Cell nuclei were counterstained with 4’,6-diami-dino-2-phenylindole (DAPI, Life Technologies). The presence of bacteria was analyzed using laser scanning confocal microscopy. Images were acquired using a confocal microscope (Zeiss LSM 800) with a 63X/1.4 oil objective, an electronic magnification of 1.0, and a resolution of 1014 × 1014 pixels.

### Inflammatory Response of Infected BMDMs and Splenocytes

For transcriptional profiling, BMDMs (5 × 10^5^ cells/well) and splenocytes (5 × 10^5^) cultured in RPMI 1640 containing 10% FBS were stimulated by viable bacteria (100 bacteria per cell, as previously reported; Fernandes et al., 2016) for 6 h, and total RNA was extracted using RNeasy Mini Kit (Qiagen) and DNAse I treatment to eliminate DNA contaminants as previously described (Mezouar et al., 2019c). The expression of genes encoding for inflammatory cytokines, microbicidal molecules, and immunoregulatory cytokines, including *IL-18*, transforming growth factor (*TGF*)-β, type-II Arginase (*Arg2*), *MCP-1*, *RANTES*, *IL-10*, *IL-12p40*, tumor necrosis factor (*TNF*), interferon (*IFN*)-γ-inducible protein-10 (*IP-10*), *perforin*, *granzyme B*, and *IL-6* was determined by qRT-PCR using specific primers (Table 1). The results were normalized to the expression of β-actin and the fold change (FC) was calculated as follows: FC = 2^–ΔΔct^, where ΔΔct = (Ct_target_ – Ct_actin_)_T–bet_ **^–/–^** – (Ct_target_ – Ct_actin_)_WT_ for splenocytes, FC = 2^–ΔΔct^, where ΔΔct = (Ct_target_ – Ct_actin_)_stimulated_ – (Ct_target_ – Ct_actin_)_unstimulated_ for each group of BMDMs. The expression of genes was considered as modulated when the FC ≥ 2.0.

**TABLE 1:** Sequences of specific primers used for real-time qRT-PCR.

Gene symbol	5′ primer (5′-3′)	3′ primer (5′-3′)
*β-actin*	TGGAATCCTGTGGCATC CATGAAACA	TAAAACGCAGCTCAG TAACAGTCCG
*IL-18*	CAGTTCCTGCCGCTGA CTAAT	CTGAGTTGCTGTTGAGAT GTGAG
*TGF-*β*1*	TGACGTCACTGGAGTT GTACG	GGTTCATGTCATGGATGGTGC
*Arg 2*	TGGATCAAACCTTGCC TCTC	GCCGATCAAATGTCTGTTCC
*MCP-1*	CTCTCTCTTCCTCCACC ACCAT	GCTCTCCAGCCTACTCATTGG
*RANTES*	CTGCTGCTTTGCCTACC TCTC	TCAGAATCAAGAAACCCTC TATCCT
*IL-10*	GTGGAGCAGGTGAAGAGT GATTT	CCAAGGAGTTGTTTCCG TTAGC
*IL-12 p40*	GACACGCCTGAAGAAG ATGAC	GCCATTCCACATGTCACTGC
*TNF*	AGAAACACAAGATGCTG GGACA	TCTGGAAAGGTCTGAAGGT AGGA
*IP-10*	CGTCATTTTCTGCCTCA TCCT	TCTGCTCATCATTCTTTTTC ATCG
*IL-6*	GGGAAATCGTGGAAA TGAGA	CTCTGAAGGACTCTGGC TTTGT
*IFN-γ*	GAGGAACTGGCAAAA GGATG	GACCTGTGCGTTGTTG ACCT
*Perforin*	CACCTCTTTCCACCAGA CCTAC	CACCGGGCTCTGCTCATT ATAT
*Granzyme B*	TAAAATGCATTCCCACC CAGA	ACACATCTCCTGGCTTC ACATT

For assessment of cytokine release, 2 × 10^6^ splenocytes cultured in RPMI 1640 containing 10% FBS were stimulated or not with heat-inactivated *C. burnetii* (10 bacteria per cell as we previously reported; Honstettre et al., 2006) for 24 h, and culture supernatants were recovered and stored at −80°C. The release of IL-6, IL-10, IFN-γ, and TGF-β1 was determined using specific immunoassays (eBioscience, France). The detection limit for the four cytokines was 6.5, 5.3, 8.6, and 15.0 pg/mL, respectively.

### Statistical Analysis

Gene expression was analyzed using the ClustVis software, and statistical analyses were performed using the GraphPad-Prism software (version 5.0). Results were analyzed with the Student *t*-test or two-way ANOVA test, and differences were considered significant when *P*-value ≤ 0.05.

## Results

### Susceptibility to *C. burnetii* Is Enhanced in T-bet^–/–^ Mice

**FIGURE 1:**
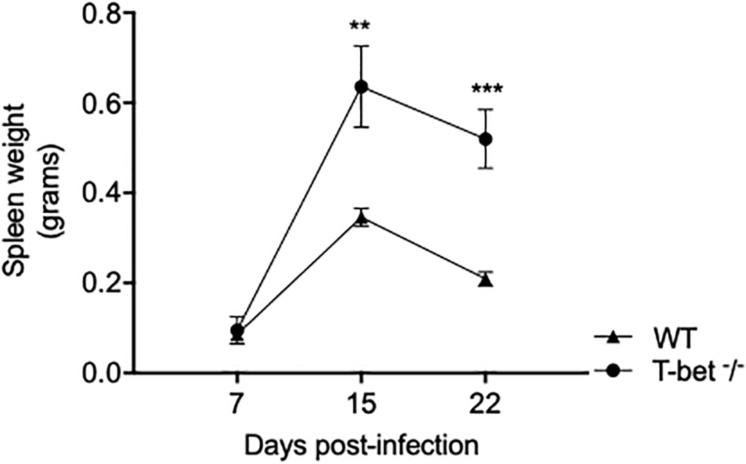
Splenomegaly in WT and T-bet^–/–^ mice after aerosol infection with *C. burnetii*. Wild-type (WT) and T-bet^–/–^ mice were infected with *C. burnetii* using an aerosol route and sacrificed at 7, 15, and 22 days postinfection. Spleen weight in grams was determined as mean ± SEM and compared using two-way ANOVA test. **P < 0.01 and ***P < 0.001 (*n* = 4 per group and for each three time points).

To assess the influence of T-bet on the susceptibility to *C. burnetii*, we first evaluated spleen weight in mice infected with *C. burnetii* as an indicator of the degree of infection. A moderate splenomegaly was detected at 7 days PI in both WT mice (0.095 ± 0.06 g) and T-bet^–/–^ mice (0.086 ± 0.007 g). Eight days later, splenomegaly dramatically increased and was significantly higher in T-bet^–/–^ mice than in WT mice (0.63 ± 0.18 g vs. 0.34 ± 0.04 g, respectively, *P* = 0.0014). Although the spleen weight was found decreased on the 22nd day PI, this latter was found significantly higher in T-bet^–/–^ mice (0.52 ± 0.13 g, *P* = 0.0007) than in WT mice (0.20 ± 0.03 g) (Figure 1), suggesting that T-bet plays a role in *C. burnetii* infection in mice.

### T-bet Is Required for Resistance to *C. burnetii* Persistence and Replication

We then determined whether the global loss of T-bet had an impact on the persistence or the elimination of *C. burnetii* by evaluating the progression of tissue infection using the qPCR.

**FIGURE 2:**
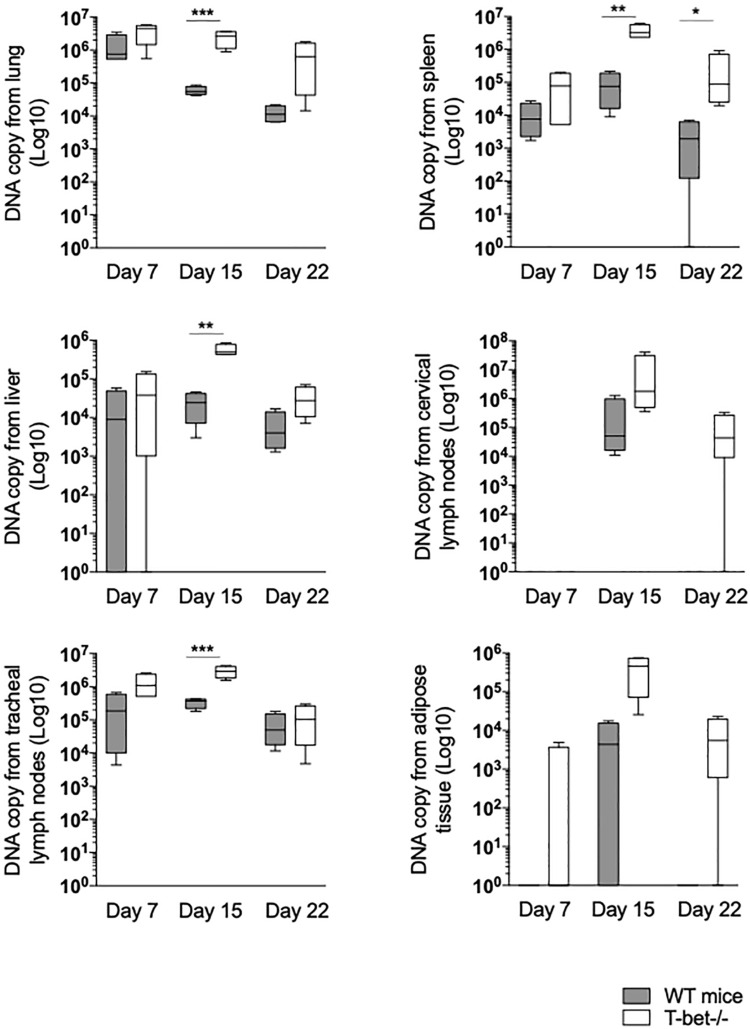
Loss of T-bet and *C. burnetii* persistence in infected mice. Wild-type (WT) and T-bet^–/–^ mice were infected with *C. burnetii* using an aerosol route. Lungs, tracheal lymph nodes, spleen, liver, cervical lymph nodes, and visceral adipose tissue were collected at 7, 15, and 22 days postinfection. Twenty-five mg of each tissue biopsy (10 mg for spleen and lymph nodes) were collected. DNA was extracted in a 100-ml volume, and the presence of *C. burnetii* DNA was assessed by qPCR using a 5 ml DNA extract. Results were expressed as DNA bacterial copy ± SD and compared using Student *t*-test. *P < 0.05, **P < 0.01, and ***P < 0.001. The samples were considered positive (cutoff value) when the qPCR Ct was <36 Ct (*n* = 4 per group and for each three time points).

First, we focused on tissues located on the entrance of *C. burnetii*, including lungs and tracheal lymph nodes (TLNs). As expected, *C. burnetii* DNA copies were detected in lungs and TLNs at a similar level at 7 days PI in WT and T-bet^–/–^ mice. In the lungs of WT mice, the number of bacterial DNA copies significantly decreased at 15 (*P* = 0.0007) and 22 days PI (*P* = 0.0001). In contrast, bacterial DNA copies persisted at 15 days PI in the lungs of T-bet^–/–^ mice and decreased thereafter but remained significantly higher than those observed in the lungs of WT mice (*P* = 0.0003 and *P* < 0.02, respectively). In TLNs, the number of *C. burnetii* DNA copies increased at 15 days PI but was significantly (*P* = 0.01) higher in T-bet^–/–^ mice than in WT mice. At day 22, a similar decrease was observed in both groups of mice (Figure 2 and Supplementary Figure S1).

Second, because local infection was observed after the aerosol route, hematogenous dissemination to immune tissues was investigated. At 7 days PI, bacterial DNA copies were found increased in lung of T-bet^–/–^ mice compared to WT mice (*P* = 0.001) (Figure 2). The number of bacterial DNA copies was slightly higher in the spleen from T-bet^–/–^ mice than in the WT mice at 15 days PI (*P* = 0.001). At 22 days PI, bacterial DNA was cleared in WT mice, whereas bacterial DNA copies were found at a similar level than at 7 days PI in T-bet^–/–^ mice (Figure 2). Additionally, no differences were observed concerning antibody titer for the phase I and phase II (Supplementary Figure S2). Livers from WT mice and from T-bet^–/–^ mice were also found positive for *C. burnetii* DNA at 7 days PI. Although bacteria were cleared thereafter in WT mice, bacterial replication in T-bet^–/–^ mice was evidenced at 15 days PI (*P* = 0.0046 between WT and T-bet^–/–^ mice) and bacterial DNA was still found at 22 days at a level similar to that detected at 7 days PI (Figure 2). We also investigated whether distal lymph nodes, such as CLNs were targeted by *C. burnetii*. At 7 days PI, copies of bacterial DNA were detected in only 1/4 of the mice in both groups. At 15 days PI, bacterial replication was observed in all (4/4) WT and T-bet^–/–^ mice. At 22 days PI, no *C. burnetii* DNA was detected in the CLNs of WT mice, whereas the number of bacterial DNA copies (3/4 mice) was still higher than that found at 7 days PI in T-bet^–/–^ mice, demonstrating that an intense bacterial replication occurred in CLNs of T-bet^–/–^ mice (Figure 2). Finally, in tracheal lymph nodes, bacterial DNA was significantly higher at 15 days PI for T-bet^–/–^ mice compared to WT mice.

Third, we tested the presence of *C. burnetii* DNA in distant tissue, such as visceral AT as we previously found targeted by *C. burnetii* (Bechah et al., 2014). Bacterial DNA was not detected in WT mice, and only 1/4 T-bet^–/–^ mouse at 7 days PI. At 15 days PI, only 2/4 WT mice were positive, whereas bacteria intensely replicated in 4/4 T-bet^–/–^mice. At 22 days PI, bacterial DNA was not evidenced in WT mice, but was still present in 3/4 T-bet^–/–^ mice (Figure 2 and Supplementary Figure S1).

**FIGURE 3:**
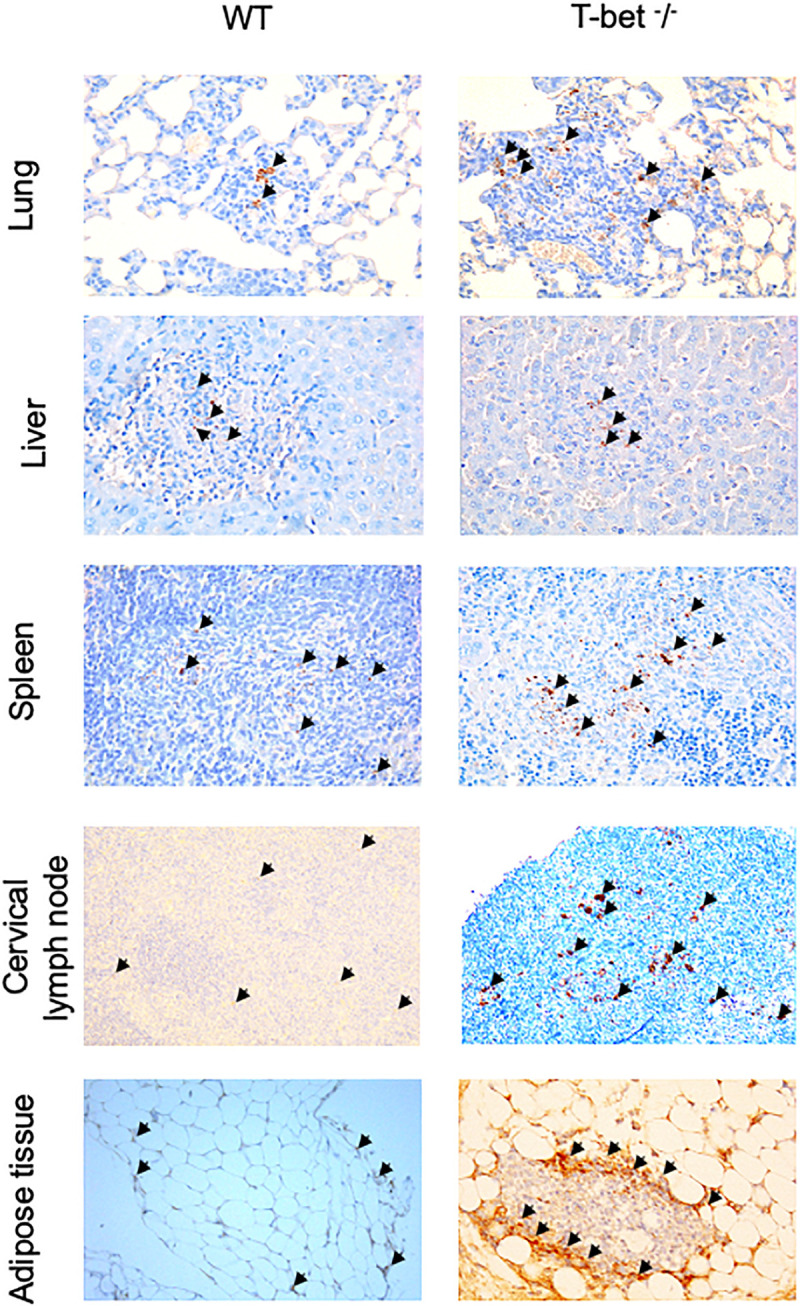
Immunohistological analysis. Wild-type (WT) and T-bet^–/–^ mice were infected with *C. burnetii* using an aerosol route. Lungs, tracheal lymph nodes, spleen, liver, cervical lymph nodes, and visceral adipose tissue were collected at 7, 15, and 22 days postinfection. Three μm sections of lungs, spleen, liver, cervical lymph nodes, and visceral adipose tissue from mice sacrificed at 15 days PI were incubated with rabbit anti-*C. burnetii* Abs. Bacteria (black arrows) were revealed using biotin-conjugated Abs and peroxidase-labeled streptavidin with amino-ethylcarbazole as substrate and appear in red. Original magnification: X100 for each micrograph (*n* = 4 per group).

Finally, we determined whether bacteria were present in these tissues using specific anti-*C. burnetii* Abs. Interestingly, we showed that *C. burnetii* was found in the investigated tissues within inflammatory lesions in WT and T-bet^–/–^ mice (Figure 3). Taken together, these results suggested that T-bet^–/–^ mice were less able to control *C. burnetii* replication and more susceptible to persistent *C. burnetii* infection than WT mice.

### Loss of T-bet Is Associated With Severe Granulomatous Lesions in *C. burnetii*-Infected Mice

**FIGURE 4:**
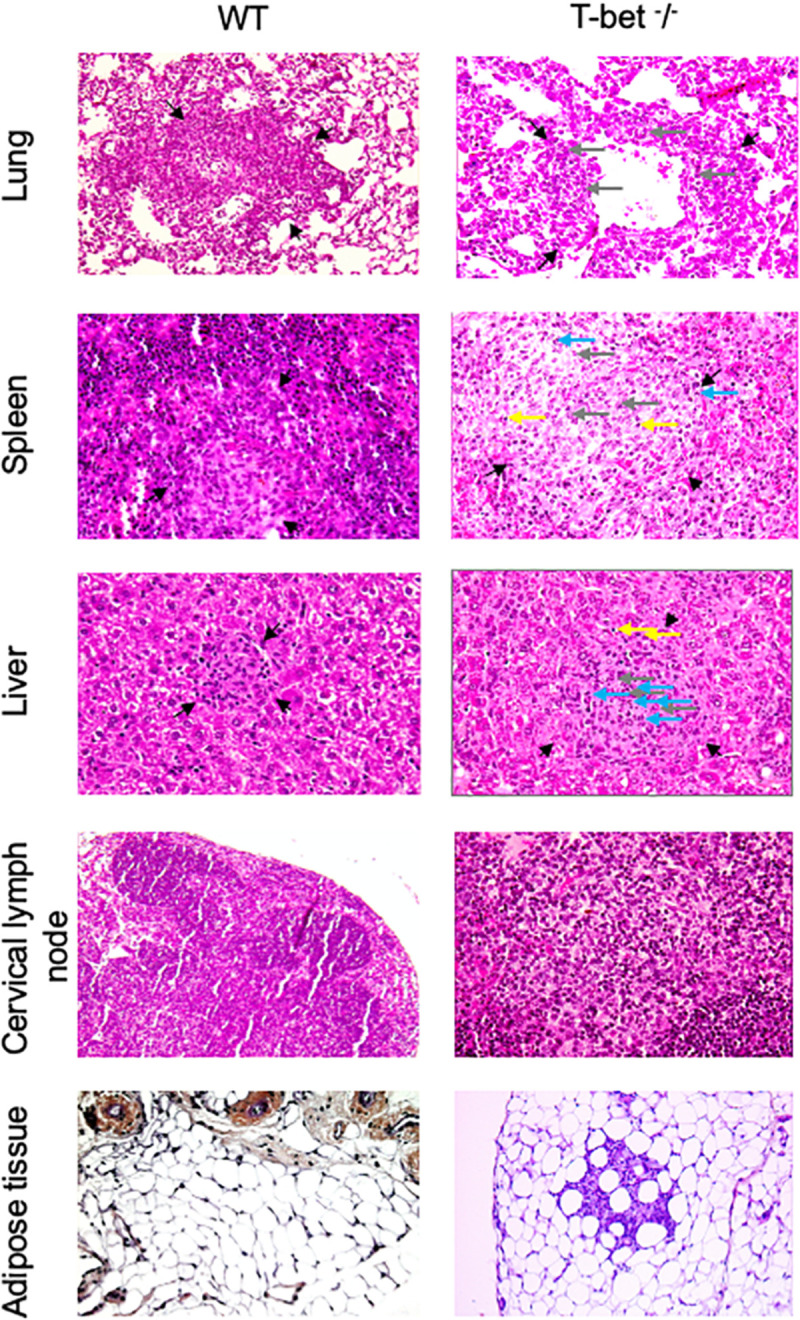
Histological analysis. Wild-type (WT) and T-bet^–/–^ mice were infected with *C. burnetii* using the aerosol route and sacrificed at 7, 15, and 22 days postinfection. Tissues were recovered, embedded in paraffin, and then sections (3 μm) were used. Sections of lung, spleen, liver, cervical lymph nodes, and visceral adipose tissue from infected-mice sacrificed at 15 days postinfection were stained with hematoxylin-eosin-saffron, and granulomatous lesions (black arrows) were observed with the original magnification: X200 for each micrograph. Gray arrows indicate macrophages, blue arrows indicate polymorphonuclear cells, and yellow arrows indicate lymphocytes (*n* = 4 per group).

As we observed inflammatory lesions using the immunochemistry approach, we next used a hematoxylin-eosin-saffron staining of tissues to evidence the presence of cell infiltration and microscopic lesions. *C. burnetii* infection induced an important macrophage infiltration in lung and numerous granulomatous lesions in the liver and spleen with similar cell composition, especially macrophages, few lymphocytes, and polymorphonuclear leukocytes in WT and T-bet^–/–^ mice (Figure 4) compared to uninfected T-bet^–/–^ mice (Supplementary Figure S3). No granulomas were evidenced in the TLNs of WT and T-bet^–/–^ mice. Surprisingly, 2/3 T-bet^–/–^ mice presented an important macrophage infiltration in CLNs and ATs at 15 days PI while WT mice had no lesions in these two tissues. Although WT mice did not exhibit lesions, 1/4 T-bet^–/–^ mice presented inflammatory lesions in visceral AT at 15 days PI and 4/4 mice at 22 days PI (Figure 4).

**FIGURE 5:**
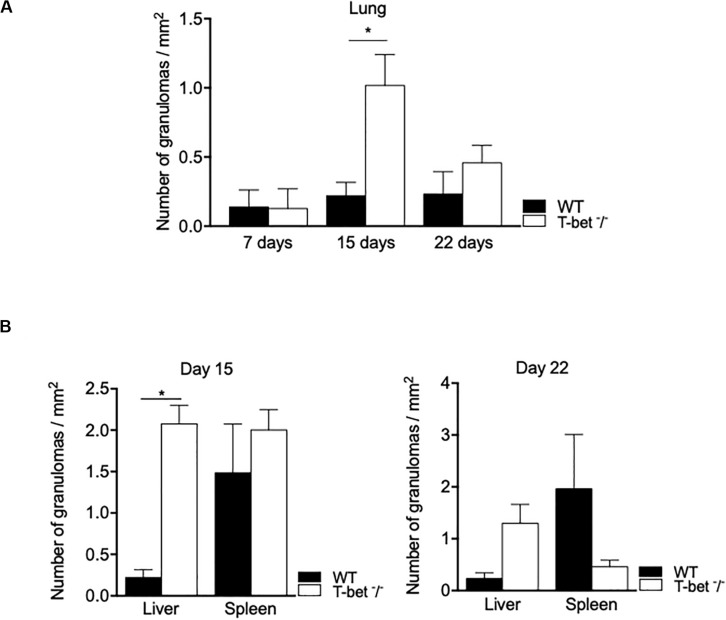
Granulomatous lesions in *C. burnetii*-infected mice. Wild-type (WT, *n* = 4) and T-bet^–/–^ (*n* = 4) mice were infected with *C. burnetii* using the aerosol route and sacrificed at 7, 15, and 22 days postinfection. Tissues were recovered, embedded in paraffin, and then sections (3 μm) were used. Granulomatous lesions defined as collections of 10 or more macrophages and lymphocytes within the biopsy sample were quantified after whole optical examination of at least three tissue sections of each organ. **(A)** The number of granuloma/mm^2^ was quantified in lungs at 7, 15, and 22 days postinfection as the mean ± SEM and compared using two-way ANOVA test. *P < 0.05. **(B)** The number of granuloma/mm^2^ was quantified in liver and spleen at 15 (left panel) and 22 (right panel) days postinfection as the mean ± SEM and compared using two-way ANOVA test. *P < 0.05. **(A,B)** Graphs at the different time points show data for all four mice per group. With the exception of the lungs at day 7 PI, only data from 3/4 mice per group that have granulomas are shown.

Focusing on the number of granulomas in lungs as a possible route of entry for *C. burnetii*, we show that it was similar in WT and T-bet^–/–^ mice at 7 days PI. At day 15 PI, the number of granulomas increased in T-bet^–/–^ mice and was significantly (*P* = 0.02) higher than in WT mice (1.01 ± 0.1 vs. 0.22 ± 0.04, respectively). At 22 days PI, the number of granulomas remained higher in T-bet^–/–^ mice than in WT mice (0.45 ± 0.06 vs. 0.23 ± 0.08) (Figure 5A).

We also observed inflammatory lesions in the immune organs, including liver and spleen at a distance from the primary infection. Granulomas were quantified at 15 days PI in the spleens of WT and T-bet^–/–^ mice and slightly decreased in T-bet^–/–^ mice at 22 days PI (Figure 5B). In contrast, the number of granulomas was significantly higher (*P* = 0.02) in the liver of T-bet^–/–^ mice than in WT mice (2.07 ± 0.1 vs. 0.37 ± 0.03) at 15 days PI. Additionally, T-bet^–/–^ mice presented macroscopic lesions at the liver surface; these same lesions were absent in WT mice (Supplementary Figure S1). At 22 days PI, the number of granulomas decreased but remained significantly higher (*P* = 0.02) in T-bet^–/–^ mice than in WT mice (0.53 ± 0.1 vs. 0.23 ± 0.05) (Figure 5B). Taken together, these results show that *C. burnetii* infection has been associated with the development of inflammatory lesions at the site of entry of bacteria and distal immune tissues with a severe granulomatous response in the lungs and liver of T-bet^–/–^ mice.

### Impaired BMDMs Immune Response in the Absence of T-bet

**FIGURE 6:**
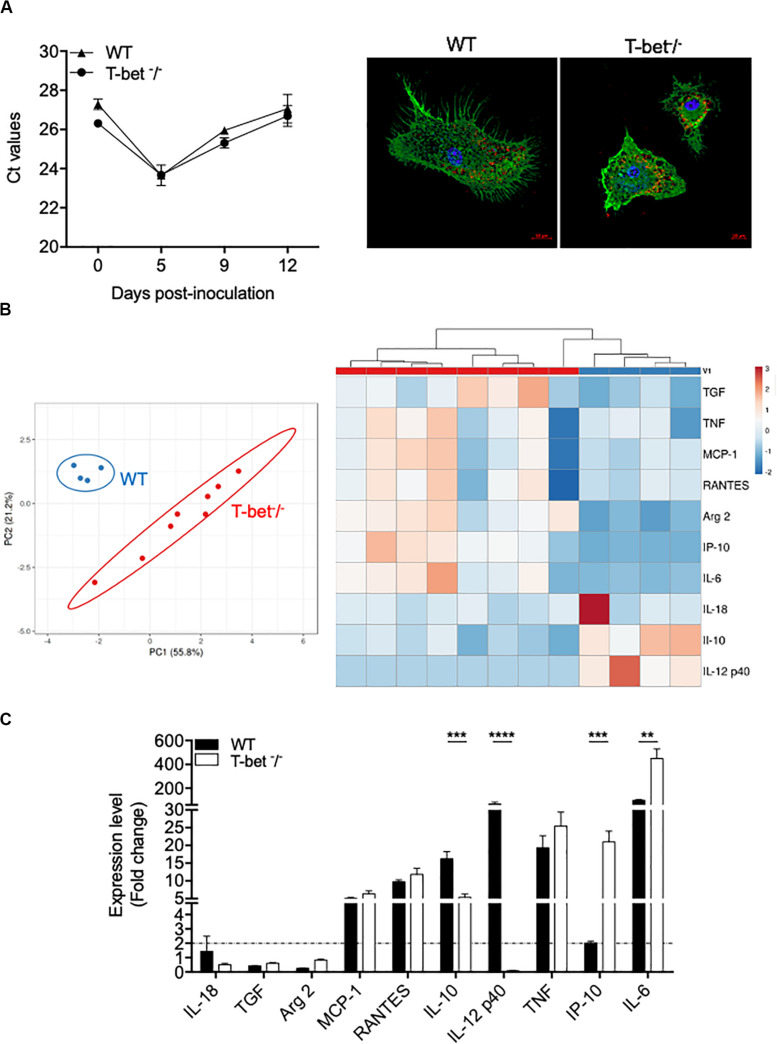
Wild-type (WT) and T-bet^–/–^-BMDMs response to *C. burnetii* infection. **(A)** WT and T-bet^–/–^ BMDMs were infected with *C. burnetii* for 4 h, washed to eliminate unbound bacteria (day 0), and cultivated for 12 days. DNA was extracted in a 100 ml volume, and the presence of *C. burnetii* DNA was assessed by qPCR using a 5-ml DNA extract. The results were expressed as mean of cycle threshold (Ct) values per 5 μl DNA extract ± SD and compared using Student *t*-test (left panel). The differences were considered significant when *P* < 0.05. Bacteria within T-bet^–/–^ BMDMs cultured on glass coverslips were labeled with rabbit Abs directed against *C. burnetii* (1:500 dilution) for 30 min and then with Alexa 555-conjugated F(ab’) anti-rabbit IgG (Molecular Probes) (1:500 dilution) and bodipy phalloidin (Alexa 488) (Invitrogen) to label filamentous actin (F-actin). Cell nuclei were counterstained with DAPI (Life Technologies). A representative microscopy micrograph obtained by confocal microscopy are shown at 5 days PI (scale bar, 10 μm) (right panel). Bacteria are shown in red, nucleus in blue, and actin in green. **(B,C)**. BMDMs were stimulated or not with *C. burnetii* for 6 h. Total RNA was extracted, and transcripts of several cytokines were quantified by qRT-PCR. Results were expressed as the ratio of expression levels in *C. burnetii-*stimulated BMDMs versus unstimulated BMDMs (*n* = 8 and *n* = 4 for T-bet^–/–^ and WT BMDMs, respectively). **(B)** A principal component analysis (left panel) and a hierarchical clustering (right panel) were realized to show the repartition of the two groups of stimulated BMDMs and the modulation of the gene expression, respectively. **(C)** Expression level of investigated genes was illustrated as the mean fold change (FC = 2^–ΔΔ^ ^ct^, where ΔΔct = (Ct_target_ – Ct_actin_)_stimulated_ – (Ct_target_ – Ct_actin_)_unstimulated_) ± SD of the fold change and compared using Student *t*-test. **P < 0.01, ***P < 0.001 and ****P < 0.0001.

As we found numerous macrophages, known as target cells for the intracellular bacterium *C. burnetii*, in granulomatous lesions, we then studied their immune response. Therefore, we incubated BMDMs from WT and T-bet^–/–^ mice with *C. burnetii* organisms (50 bacteria per cell) for 4 h (day 0). Cells were washed to eliminate unbound bacteria and cultured for 12 additional days. No significant differences were observed concerning the uptake and the survival of *C. burnetii* organisms by BMDMs in T-bet^–/–^ mice compared to WT mice (Figure 6A). Interestingly, at 5 days PI, the bacteria replicated in both BMDMs groups in the same way, and the number of bacterial DNA copies decreased in a similar way in WT and T-bet^–/–^ mice at 9 and 12 days PI, but bacteria survived (Figure 6A). These results show that *C. burnetii* transiently replicated within BMDMs but that T-bet was not involved in the intracellular life of *C. burnetii*.

Thereafter, we wondered whether *C. burnetii* stimulates different transcriptional patterns in BMDMs according to the expression of T-bet. WT and T-bet^–/–^ BMDMs were stimulated with *C. burnetii* organisms (100 bacteria per cell) for 6 h, and the expression of selected genes was then assessed. Using a principal component analysis approach, we showed a clear separate response of stimulated BMDMs according to the investigated group (Figure 6B). Interestingly, hierarchical clustering analysis revealed a different immune response between WT and T-bet BMDMs. First, the genes encoding *IL-18*, *TGF-*β, and *Arg2* were not expressed in WT and T-bet^–/–^ BMDMs (Figure 6C). Second, the expression of genes encoding *TNF*, *RANTES*, and MCP-1, two chemokines, was induced in BMDMs independently of T-bet. Third, the gene encoding *IL-12 p40* was expressed in WT BMDMs but not in T-bet^–/–^ BMDMs (*P* = 0.0001). Fourth, the expression of the *IL-10* gene was threefold lower in T-bet^–/–^ BMDMs than in WT BMDMs (*P* = 0.0002). Conversely, the expression of genes encoding *IP-10* (*P* = 0.001) and *IL-6* (*P* = 0.01) was significantly 10- and 4.5-fold higher in T-bet^–/–^ BMDMs than in WT BMDMs, respectively (Figure 6C). Taken together, these results demonstrate that the absence of T-bet substantially affects the role of macrophages against *C. burnetii*.

### The Absence of T-bet Leads to an Altered Antimicrobial Response of Splenocytes

**FIGURE 7:**
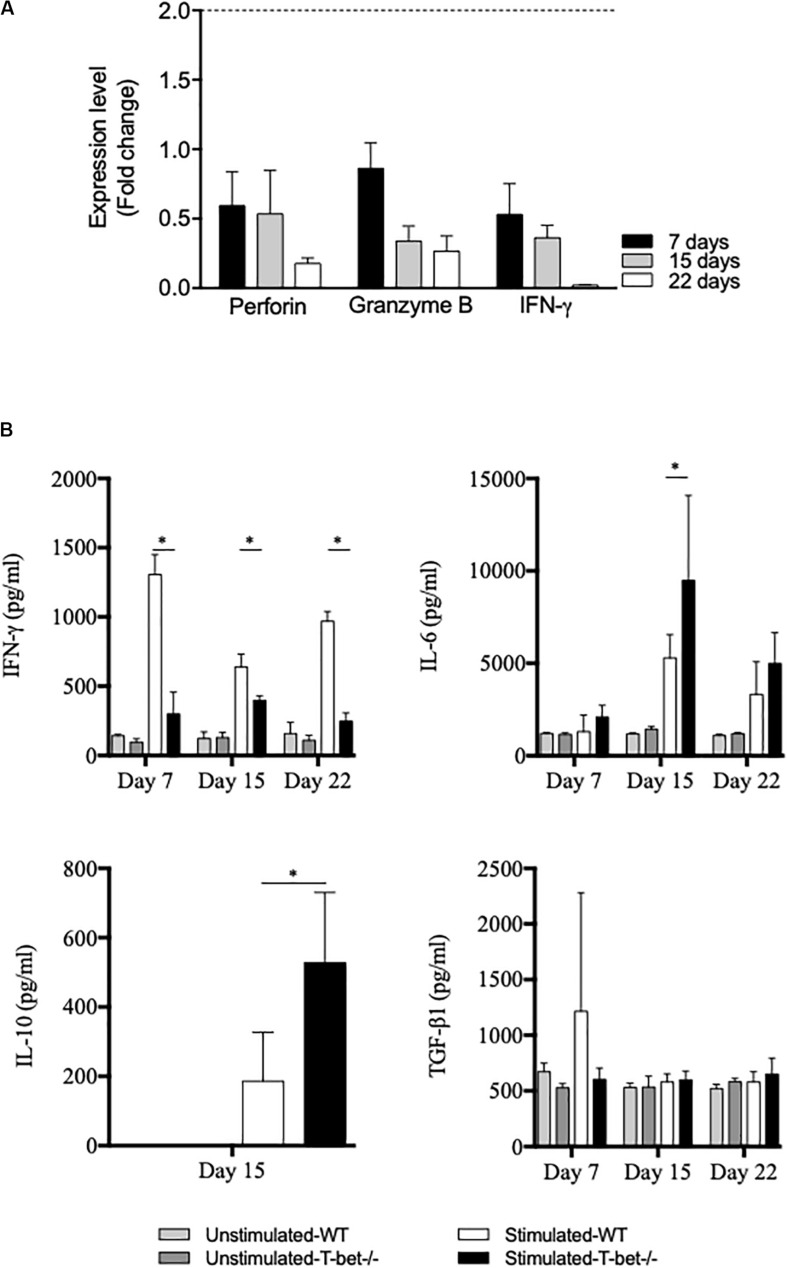
Antimicrobial and inflammatory responses of infected splenocytes. Isolated splenocytes from 4 WT and 4 T-bet^–/–^ infected-mice were collected at 7, 15, and 22 days. **(A)** The expression of genes encoding *perforin*, *granzyme B*, and *IFN-*γ was quantified using qRT-PCR. The results were expressed as the ratio of expression (fold change) levels in T-bet^–/–^ splenocytes versus WT splenocytes and the mean fold change ± SD was considered as modulated when the FC ≥ 2.0 [FC = 2^–ΔΔ^ ^ct^, where ΔΔct = (Ct_target_ – Ct_actin_)_T–bet_^–/–^ – (Ct_target_ – Ct_actin_)_WT_]. The differences were considered significant when *P* < 0.05. **(B)** Isolated splenocytes were incubated in the presence or absence of heat-inactivated *C. burnetii* (10 bacteria per cell) for 24 h. Supernatants were sampled, and the production of IFN-γ, IL-6, IL-10, and TGF-β1 was determined by immunoassays as mean ± SEM and compared using two-way ANOVA test, ^∗^*P* < 0.05.

Because the absence of T-bet increased *C. burnetii* replication and persistence in the spleen, we investigated the expression of genes known to be involved in the microbicidal response, including *granzyme B*, *perforin*, and *IFN-*γ by splenocytes. Splenocytes from infected mice were incubated with bacteria (100 bacteria per cell) for 6 h, and gene expression was assessed by the evaluation of the fold change (Figure 7A). First, the investigated genes were down-modulated in T-bet**^–^**/**^–^** mice compared to WT mice. Second, as the expression of the gene encoding *IFN-*γ was also found down-modulated in T-bet**^–^**/**^–^** mice, its production by infected-splenocytes was then assessed (Figure 7B). The spontaneous production of IFN-γ by WT and T-bet**^–^**/**^–^** splenocytes was similar, but after stimulation with heat-inactivated *C. burnetii*, T-bet**^–^**/**^–^** splenocytes released significantly lower levels of IFN-γ than WT (*P* < 0.003). In contrast, the release of immunomodulatory cytokines, such as IL-6 and IL-10, was significantly increased in T-bet**^–^**/**^–^** splenocytes compared to WT splenocytes at day 15. No IL-10 level was detected at days 7 and 22 (data not show). Additionally, no significant differences were observed for the release of TGF-β1 in WT and T-bet**^–^**/**^–^** splenocytes of each kinetic tested (Figure 7B). Collectively, these results indicate that the absence of T-bet is associated with an altered antimicrobial response and immunoregulatory cytokines production by splenocytes stimulated with *C. burnetii*.

## Discussion

It has been suggested, at least in mice, that IFN-γ-mediated immunity is essential to protect the host from *C. burnetii* infection as supported by the high mortality observed in IFN-γ**^–^**/**^–^** mice infected with *C. burnetii* (Andoh et al., 2007). Moreover, *C. burnetii*-specific IFN-γ is produced by PBMCs from subjects vaccinated against *C. burnetii* and after natural infection (Izzo and Marmion, 1993). However, in humans, the persistence of *C. burnetii* infection does not appear to be associated with deficiency in IFN-γ production (Schoffelen et al., 2013, 2017; Keijmel et al., 2016). Therefore, the production of IFN-γ alone does not explain the failure to clear the *C. burnetii* infection. IFN-γ is under the control of type I interferons and IL-12, IL-18, and IL-23 (van de Vosse et al., 2009). Other cytokines also play a role in Q fever as TNF-α, IL-1-β, and IL-10 as well as IL-2 and the IL-12/IFN pathway does play a role (Schoffelen et al., 2017). Here, we report that loss of T-bet confers increased susceptibility to *C. burnetii* infection. Indeed, T-bet-deficient mice infected with *C. burnetii* by aerolization presented an increase in persistent infection and bacterial replication in the respiratory tract (lungs and tracheal lymph nodes), immune system (spleen and liver), and distant tissues (visceral adipose tissue) compared to WT mice. Using a histological approach, we showed an increase in the number of granulomas in liver, lungs, and spleen of T-bet**^–^**/**^–^** mice. Moreover, only T-bet**^–^**/**^–^** mice present inflammatory lesions in distant tissues, including adipose tissue and cervical lymph nodes, suggesting that an uncontrolled immune response was associated with infection and its dissemination. Our results show, despite T-bet-associated immune alteration, T-bet**^–^**/**^–^** mice maintain their ability to control infection after 3 weeks of infection. T-bet**^–^**/**^–^** mice inoculated intravenously with *Staphylococcus aureus* lead to 20% mortality at 10 days after inoculation (Hultgren et al., 2004). In contrast, airborne infection with *Mycobacterium tuberculosis* induced only 1/5 T-bet**^–^**/**^–^** mouse survival at 100 days PI, whereas no WT mouse succumbs (Sullivan et al., 2005). Here, we did not observe mortality in T-bet**^–^**/**^–^** mice infected with Guiana strain described as the most virulent *Coxiella* strain. Further investigations are required to explore the role of T-bet during *C. burnetii* over extended periods of time and to study the response to other strains of *Coxiella*.

T-bet, which is a central regulator of Type 1 immunity, is essential for defense against infection by intracellular pathogens as demonstrated by loss of function models. Indeed, it has previously been reported that T-bet-deficient mice are unable to control bacterial replication following infection with intracellular pathogens, such as *Salmonella enterica serovar typhimurium* (Ravindran et al., 2005), *Leishmania major* (Szabo, 2002), *Francisella tularensis* (Melillo et al., 2014), or *Mycobacterium avium* and *tuberculosis* strains (Sullivan et al., 2005; Matsuyama et al., 2014). Here, we found that the absence of T-bet increased the bacterial load locally, including TLN and lungs, and at distal sites, including visceral AT and CLN, demonstrating a spread of infection. Thus, we provide evidence that T-bet was also involved in the regulation of infection and the persistence of the intracellular bacterium *C. burnetii*. This finding is in accordance with previous studies indicating that the *in vitro* persistence of *C. burnetii* infection or the fatal outcome of *C. burnetii* infection in animal model is associated with altered Th1 immune response (Dellacasagrande et al., 1999; Andoh et al., 2007), thus opening up new approaches to understanding the pathogenesis of this infectious disease.

Here, we report that infected-T-bet**^–^**/**^–^** mice presented increased numbers of inflammatory lesions characteristic of granulomas in the investigated tissues. These observations could be explained by an increase in proinflammatory cytokines, known to be involved in the formation of granulomas, such as TNF that we found up-modulated in T-bet**^–^**/**^–^** mice. Indeed, it has been shown that TNF neutralization of targeted mutation resulted in a loss of granuloma number and structure (Algood et al., 2005). Here, these granulomatous lesions were organized and were rich in macrophages with few lymphocytes and polymorphonuclear leukocytes (Figure 4) as observed in Q fever endocarditis (Capo and Mege, 2012), thus suggesting that the loss of T-bet did not disturb the cellular composition of lesions. Thus, although the protective role of tuberculous granuloma is well documented (Carow et al., 2019), that of *Coxiella* granuloma remains obscure. Analyses complementary to those carried out here by histology should make it possible to evaluate more precisely the role of the *Coxiella* granuloma.

The effect of T-bet was specific. Indeed, in our experimental conditions, loss of T-bet confers increased susceptibility to *C. burnetii* infection. However, T-bet**^–^**/**^–^** BMDM infected by *C. burnetii* present a similar profile in terms of entry and the outcome of bacteria as compared to WT mice. As T-bet acts specifically on the Th1 response, it is likely that T-bet affects the outcome of a *C. burnetii* infection via the host’s adaptive immune response rather than via the innate response. In contrast, there’s a different transcriptional immune response, including a decrease in IL-10 expression, while IL-6 and IP-10 have increases in T-bet**^–^**/**^–^** BMDM as compared to WT BMDM. This configuration was previously reported in *C. burnetii* infected-BALB/c mice (Schoffelen et al., 2015). Indeed, BALB/c mice infected with *C. burnetii* via the aerosol route presented an increased production of proinflammatory cytokines, including IL-6 and IP-10 and a very low release of IL-10. These results are consistent with our previous observations that show an increase in these cytokines in patients with acute Q fever (Honstettre et al., 2003). This specific immune response warrants further investigation, in particular to understand the role of these cytokines in the control of BMDM activity or that of the T lymphocyte population.

We hypothesized that the absence of T-bet increases *C. burnetii* infection and its persistence by altering the lymphoid compartment. Using isolated splenocytes, the absence of T-bet was associated with impaired IFN-γ production; the principal protective cytokine released by Th1 effector CD4^+^ T cells, and increased the production of IL-6 and IL-10, two immunoregulatory cytokines. Thus, the imbalance between inflammatory and immunoregulatory cytokines may decrease the expression of two microbicidal genes, such as those encoding granzyme B and perforin 1. We found that these genes were down-modulated in T-bet**^–^**/**^–^** splenocytes. It is likely that the T-bet deficiency contributes to the defective control of *C. burnetii* infection through its effect on the lymphoid compartment. T-bet was originally described as a transcription factor crucial in initiating Th1 lineage development from naïve T cells (Szabo et al., 2000) able to reprogram fully polarized Th2 cells into Th1 phenotype (Lametschwandtner et al., 2004) and to impede the development of Th17, a population of effector CD4^+^ T cells (Yeh et al., 2014). Preliminary data from our team show that peripheral blood mononuclear cells from patients with persistent Q fever produce less IL-17 and IL-17RA compared to patients with acute Q fever (unpublished data). Recently, several studies have shown that other lymphoid cells, including CD8 + T cells, B cells, and some innate lymphoid cells (ILCs), require T-bet for their development and/or differentiation (Kallies and Good-Jacobson, 2017). Indeed, group 1 of these innate lymphoid cells, called ILC1s, in addition to the expression of IL-7R at their surface as the other ILCs, the ILC1 subsets expressed T-bet, which is required for their differentiation, and these cell subsets produce the IFN-γ, Th1 cytokine required to control intracellular infections as demonstrated in the case of infection with *Toxoplasma gondii* (Klose et al., 2014). The contribution of ILC-1 in *C. burnetii* infection needs to be further investigated. It has also been demonstrated that T-bet was expressed in epithelial cells of the human female reproductive tract under the regulation of female steroid hormones. This expression involves female steroid hormone/Stat 1 and Stat 5/T-bet/IL-15 pathway unlike the T-bet pathway in lymphoid cells regulated by Stat 1/4 and IFN-γ (Kawana et al., 2005). All these aspects of T-bet should be investigated in further studies using animal models of *C. burnetii* infection.

Our results highlight the formation of granulomas in a context of low-diffusion IFN-γ release suggesting a different mode of granuloma formation during *C. burnetii* infection. Using an *in vitro* model of granuloma formation, we previously reported that the transcriptional response of *C. burnetii*-generated granulomas was associated with a down-modulation of genes encoding for T-bet, including IFN-γ (Faugaret et al., 2014). However, the essential role of IFN-γ in granuloma formation is well documented in tuberculosis infection (Asano et al., 1993; Hogan et al., 2001). This raises questions about the mechanisms of formation and the function of granuloma formation in *C. burnetii* infection.

## Conclusion

In conclusion, we demonstrated that T-bet**^–^**/**^–^** mice were more affected than WT mice by *C. burnetii* infection as evidenced by defective bacterial control, persistent infection, and organ injury. The absence of T-bet has also been associated with an increased production of immunomodulatory cytokines, an impaired production of IFN-γ and the expression of microbicidal genes, suggesting that T-bet controls *C. burnetii* infection in mice through the modulation of the specific immune response. Finally, this study reinforces the previous observations illustrating an alteration of the Th1 pathway by *C. burnetii* and opens new perspectives in the comprehension of the Q fever disease.

## Data Availability Statement

The datasets generated for this study are available on request to the corresponding author.

## Ethics Statement

The animal study was reviewed and approved by the experimental protocol (reference APAFIS #2016101209473180) and Ethics Committee “C2EA-14,” of Aix-Marseille University, Marseille, France, and the French Ministry of National Education, Higher Education and Research.

## Author Contributions

SM, HL, IO, and YB performed the experiments and analyzed the data. J-PG provided access to deficient mice. SM, DR, J-LM, and YB supervised the work. YB conceived and supervised animal experimental work. SM and YB wrote the manuscript. All authors read and approved the final manuscript.

## Conflict of Interest

The authors declare that the research was conducted in the absence of any commercial or financial relationships that could be construed as a potential conflict of interest.

## Supplementary Material

FIGURE S1 Macroscopic lesions of liver from infected mice. WT and T-bet^–/–^ mice were infected with *C. burnetii* using the aerosol route and sacrificed at 15 days PI. The presence of lesions was investigated macroscopically.

FIGURE S2 Antibody response in mice after *C. burnetii* infection. WT and T-bet^–/–^ mice were infected with *C. burnetii* using the aerosol route and sacrificed at 7, 15, and 22 days PI. The titer of specific Abs directed against *C. burnetii* phase I and phase II was assessed by immunofluorescence assay. The results are expressed as median ± SD of four mice per time point.

FIGURE S3 Histological and immunochemistry study in uninfected T-bet^–/–^ mice. Tissues were recovered, embedded in paraffin, and then sections (3 μm) were used. **(A)** Sections of lung, liver, spleen, cervical lymph nodes, and visceral adipose tissue from uninfected mice were stained with hematoxylin-eosin-saffron, and the presence of lesions were examined with the original magnification: X200 for each micrograph, X300 for lymph nodes. **(B)** Three μm sections of these different tissues were incubated with rabbit anti-*C. burnetii* Abs. The presence of bacteria was revealed using biotin-conjugated Abs and peroxidase-labeled streptavidin with amino-ethylcarbazole as substrate and appear in red. Original magnification: X100 for liver and spleen, X300 for lung, lymph nodes, and adipose tissue.
