# Supplementary figures and images for: T-Bet Controls Susceptibility of Mice to Coxiella burnetii Infection
Source: Front Microbiol. 2020 Jul 14;11:1546. doi: 10.3389/fmicb.2020.01546 (PMC7381240; doi:10.3389/fmicb.2020.01546)

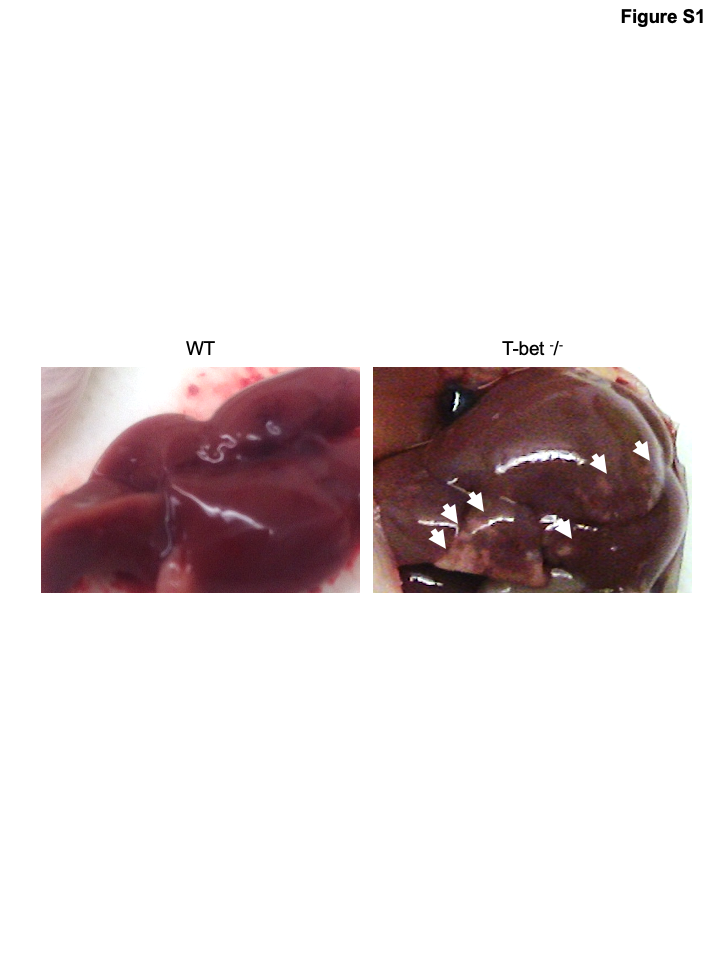

Supplement: FIGURE S1 — Macroscopic lesions of liver from infected mice. WT and T-bet–/– mice were infected with C. burnetii using the aerosol route and sacrificed at 15 days PI. The presence of lesions was investigated macroscopically. [file Image_1.TIFF]

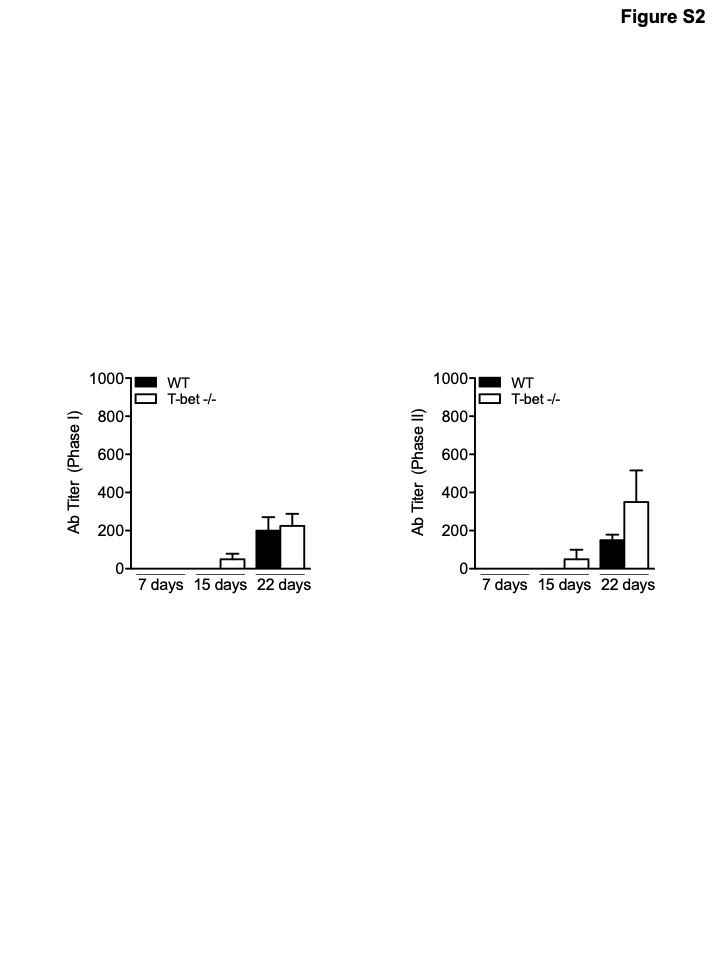

Supplement: FIGURE S2 — Antibody response in mice after C. burnetii infection. WT and T-bet–/– mice were infected with C. burnetii using the aerosol route and sacrificed at 7, 15, and 22 days PI. The titer of specific Abs directed against C. burnetii phase I and phase II was assessed by immunofluorescence assay. The results are expressed as median ± SD of four mice per time point. [file Image_2.TIFF]

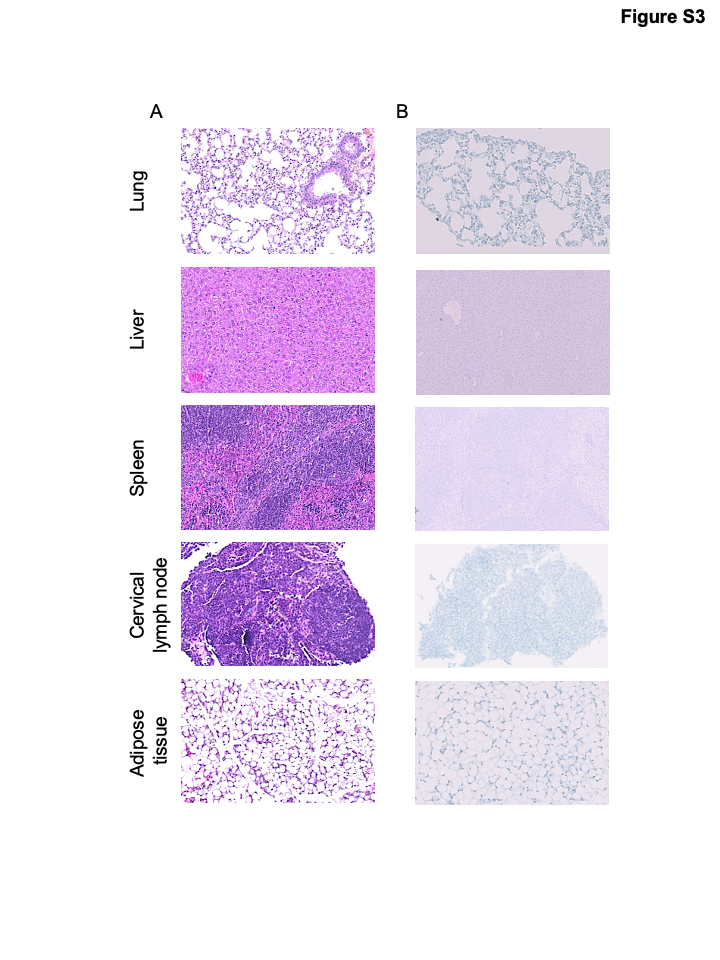

Supplement: FIGURE S3 — Histological and immunochemistry study in uninfected T-bet–/– mice. Tissues were recovered, embedded in paraffin, and then sections (3 μm) were used. (A) Sections of lung, liver, spleen, cervical lymph nodes, and visceral adipose tissue from uninfected mice were stained with hematoxylin-eosin-saffron, and the presence of lesions were examined with the original magnification: X200 for each micrograph, X300 for lymph nodes. (B) Three μm sections of these different tissues were incubated with rabbit anti-C. burnetii Abs. The presence of bacteria was revealed using biotin-conjugated Abs and peroxidase-labeled streptavidin with amino-ethylcarbazole as substrate and appear in red. Original magnification: X100 for liver and spleen, X300 for lung, lymph nodes, and adipose tissue. [file Image_3.TIFF]
